# Supplementary material for: Modification of i-GONAD Suitable for Production of Genome-Edited C57BL/6 Inbred Mouse Strain
Source: Cells. 2020 Apr 13;9(4):957. doi: 10.3390/cells9040957 (PMC7226992; doi:10.3390/cells9040957)
Supplement: Supplementary file 1 [file cells-09-00957-s001.pdf]

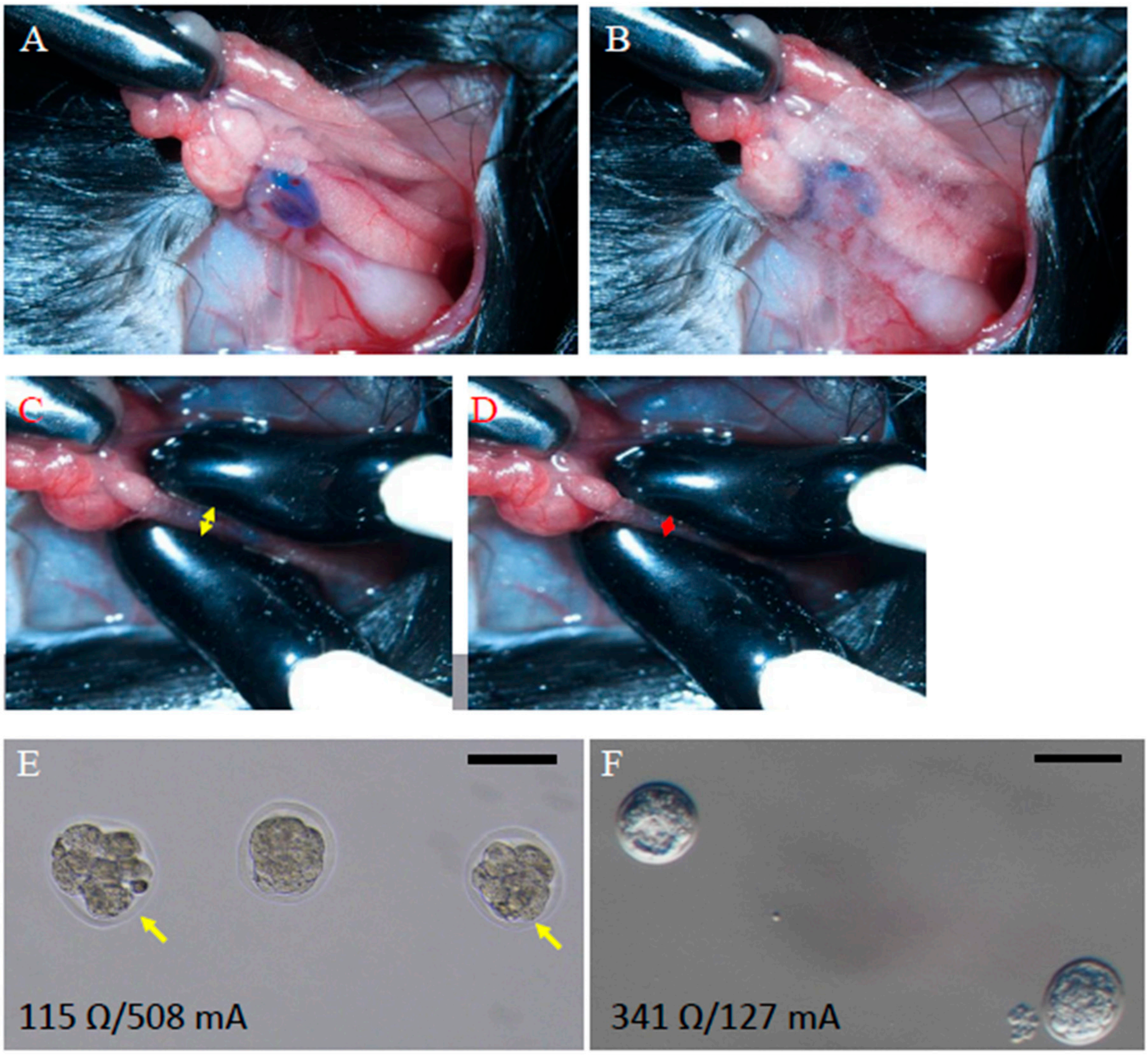

Figure S1. Adjustment of the distance between the two electrodes to obtain optimal current (**A**–**D**) and abnormal embryos (**E**,**F**), probably caused by a high current. **A**. The exposed ovary, oviduct, and part of the uterus before *i*-GONAD. After placing these on the back skin of mice, adipose tissue was anchored with an Aorta-Klemme to prevent return of the exposed tissues. Then, CRISPR reagents together with blue dye were injected into the oviductal lumen. **B.** The oviduct covered with a small piece of wet KimWipe towel. **C.** The oviduct sandwiched by the tweezer-type electrodes. The distance between the two electrodes is about 2 mm in length (shown by yellow arrows). **D.** The oviduct sandwiched by the tweezer-type electrodes. The distance between the two electrodes is about 1 mm (shown by red arrows). **E.** Embryos (corresponding to the morula stage) collected 2 days after *i*-GONAD performed under high current conditions (40 V/115 Ω/508 mA). Some embryos (shown by arrows) died or underwent developmental arrest. **F.** Embryos (corresponding to the morula stage) collected 2 days after *i*-GONAD performed under optimal conditions (40 V/341 Ω/127 mA). Each embryo exhibited normal morphology. Bar: 100 μm.
